# Acute Spontaneous Bilateral Adrenal Hemorrhage Presenting as Hyponatremia

**DOI:** 10.7759/cureus.33060

**Published:** 2022-12-28

**Authors:** Venu Madhav Chippa, Swetha Chenna, Rahul Gujarathi, Narsimha Candula

**Affiliations:** 1 Internal Medicine, St Vincent medical center, Evansville, USA; 2 Internal Medicine, Indiana University, Indianapolis, USA; 3 Hospital Medicine, University of Florida Health, Jacksonville, USA; 4 Hospital Medicine, University of Florida College of Medicine, Jacksonville, USA

**Keywords:** confusion, hypotension, hyponatremia, adrenal insufficiency, adrenal hemorrhage

## Abstract

Bilateral adrenal hemorrhage is a very unusual cause of severe adrenal insufficiency and hyponatremia. It can result from trauma, infections, or antiphospholipid antibody syndrome and can be fatal if not diagnosed and treated early. Here, we present a 58-year-old Caucasian man with fatigue, altered sensorium, bradycardia, and hypotension. He denied any abdominal pain, recent trauma, or anti-platelet or anti-coagulation agents. His laboratory workup showed hyponatremia with low serum cortisol levels. He was further worked up and underwent computerized tomography (CT) of the abdomen, which showed bilateral adrenal hemorrhage. He was treated with intravenous (IV) steroids followed by oral hydrocortisone and fludrocortisone. His symptoms resolved, and he was safely discharged home. Asymptomatic bilateral adrenal hemorrhage is a sporadic disease, and it should be in the differential diagnosis for disproportionately sick people with other adrenal insufficiency features.

## Introduction

Bilateral adrenal hemorrhage is a rare cause of acute adrenal insufficiency. It is a medical emergency and can be fatal. Bilateral adrenal hemorrhage was identified in at least 15% of people who died of shock on autopsy [1]. The estimated prevalence of primary adrenal insufficiency is between 4-60 per million [2]. The data on mortality in adrenal insufficiency is not very clear, but studies suggest that there may be a twofold increase compared to the general population. The incidence of acute adrenal crisis in patients with adrenal insufficiency in Europe is around 6-8 per 100 patient-years. The mortality rate is around 0.5 per 100 patient-years. The long-term morbidity of adrenal insufficiency is not well-established [3]. Clinically profound adrenal insufficiency occurs only after 90% of the adrenal cortex is destroyed [4].

Common adrenal hemorrhage symptoms are vague and include abdominal pain, fatigue, malaise, and hypotension. It is commonly seen in older men with a mean age of 61. Adrenal hemorrhage can be classified into traumatic and nontraumatic. The most common nontraumatic causes are anti-coagulation (heparin, coumadin) therapy, postoperative, sepsis, meningococcal infection (Waterhouse Friderichsen syndrome), antiphospholipid antibody syndrome, Sunitinib, Human Immunodeficiency Virus, Cytomegalovirus, and fungal infections [5]. In young people, it is commonly associated with antiphospholipid antibody syndrome. Spontaneous adrenal hemorrhage is bleeding in the adrenal gland, and the absence of trauma, anticoagulation, or any other underlying adrenal pathology, and its prevalence is unknown.

During stress, there is an increased amount of catecholamines, causing vasoconstriction of the adrenal vein in venules. This in turn causes adrenal vein thrombi, and the subsequent increased adrenal venous pressure leads to rupture, resulting in adrenal hemorrhage. The mechanism of action of adrenal insufficiency and bilateral adrenal hemorrhage is because of infarction secondary to loss of blood supply [6]. Hyponatremia is serum sodium less than 135 mmol/L and is the most common electrolyte abnormality in clinical practice worldwide. The most common causes of hyponatremia are the syndrome of inappropriate antidiuretic hormone (SIADH), diuretic use, polydipsia, hypovolemia, adrenal insufficiency, liver cirrhosis, and heart failure. As age is a strong independent risk factor for hyponatremia, it is relatively more common in older adults than in young adults. The common hyponatremia symptoms are nausea, vomiting, headache, fatigue, cognitive impairment, falls, and gait disturbances. Severe symptoms include stupor, coma, and seizures [7]. The prevalence of hyponatremia in the normal population is around 8%, and it increases significantly with age [8]. Aging also impairs water excretion capacity. The lack of specific symptoms and definitive examination findings makes this diagnosis challenging. Providers should have a very high index of suspicion for adrenal hemorrhage in elderly with severe hyponatremia, hypotension, and confusion. Early detection and rapid treatment will help save lives. They need appropriate follow-up and long-term management with steroids.

## Case presentation

A 58-year-old Caucasian male presented to our emergency department with complaints of generalized weakness and altered sensorium for four days associated with decreased oral intake. In the emergency room, he was accompanied by his daughter, who is the primary source of information. He never had similar complaints in the past. He denies any fever, headache, chest pain, dizziness, abdominal pain, or a recent change of medications. His medical history is significant for hyperlipidemia, hypertension, and peripheral artery disease requiring an aortoiliac stent. He takes metoprolol, atorvastatin, hydrochlorothiazide, and aspirin. He is an active smoker and drinks about 5-6 beers daily. In the emergency room, his vitals are as follows: temperature of 37.2 °C, heart rate of 76 bpm, respiratory rate of 16 breaths/min, blood pressure of 96/67 mm of Hg, and saturation of 94% on room air. On examination, he is well developed and appears in no distress, and his skin is warm and dry; pupils are equally reactive to light with pale conjunctival mucosa, normocephalic atraumatic head, supple neck, no thyromegaly, normal heart and lung sounds, and the abdomen is soft, non-tender, with normal bowel sounds. He is drowsy, responds to verbal stimuli with a short attention span, and dozes off in between; he can move all four extremities and has normal reflexes and sensations with Glasgow come scale of 12, E(3), V(3) and M(6).

Initial laboratory work-up showed elevated white blood cells of 16,400/mm³, creatinine 1.2 mg/dL, and lactic acid 4.5 mmol/L. His serum sodium was 129 mmol/L, potassium levels were 4.2 mmol/L (normal range 3.6 - 5.2 mmol/L), serum osmolality was 267 mOsm/kg, and urine sodium was 28 meq/L. Blood alcohol levels were < 10 mg/dL. Random cortisol levels were < 1 mcg/dL (Normal range 3.7 to 19.4 mcg/dL), and his blood aldosterone was < 1 ng/dL (normal range 3 to 16 ng/dL supine position). His other labs including hemoglobin, hematocrit, platelets, potassium, chloride, bicarbonate, glucose, blood urea nitrogen, calcium, creatinine phosphate kinase, troponins, urinalysis, anticoagulation panel, and thyroid function tests were within normal limits. 

**Figure 1:**
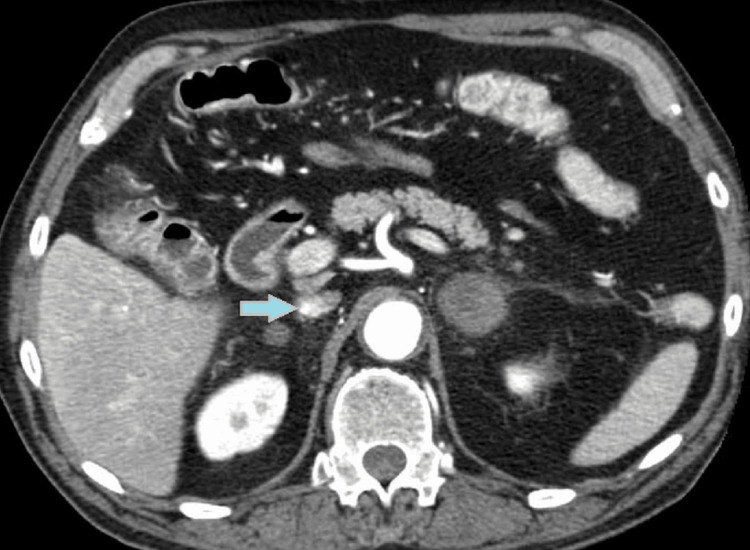
CT scan of the abdomen showing right adrenal hemorrhage (Blue arrow with yellow margin).

An emergent head, chest, and abdomen CT scan without contrast was done in the emergency room because of hypotension, severe hyponatremia, and elevated lactic acid. CT of the abdomen showed heterogeneous low-attenuation right adrenal nodularity with adjacent stranding suspicious for adrenal hemorrhage and a patent aortoiliac stent (Figure 1). 

**Figure 2:**
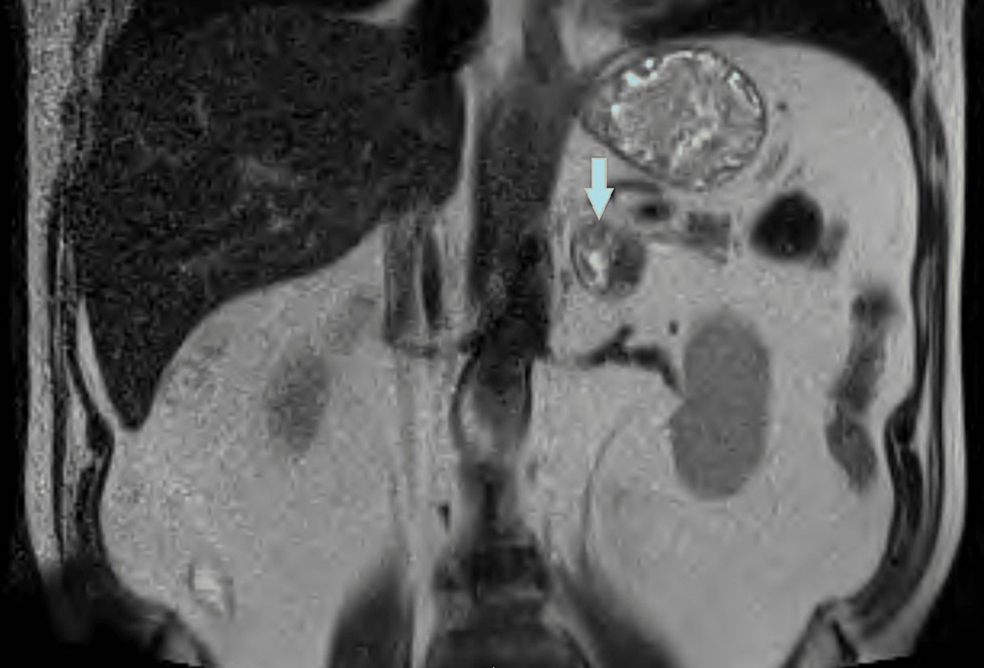
MRI of the abdomen (coronal section) showing left adrenal gland hemorrhage (Blue arrow with yellow margin).

**Figure 3:**
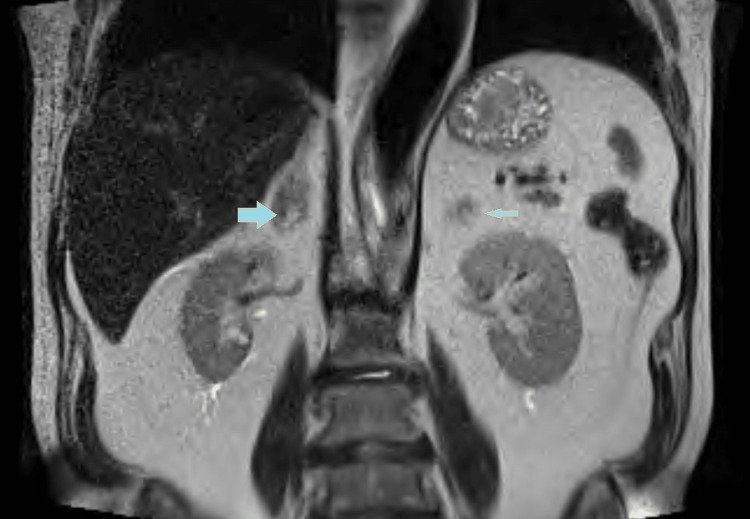
MRI of the abdomen (coronal section) showing right and left adrenal gland hemorrhage (Blue arrow with yellow margin).

Once the patient was stable, an MRI of the abdomen with and without contrast was done on day three, showing heterogeneous mixed-signal nodularity in both adrenal glands measuring 3.3x2.7 cm on the left and 2.3x1.8 cm on the right side, suggesting bilateral adrenal hemorrhages (Figure 2 and 3).

After receiving 2 L of normal saline bolus, he received 100 mg of IV hydrocortisone followed by 50 mg every 6 hours. His four-hour follow-up serum lactic acid improved. We continued to hold his hydrochlorothiazide, metoprolol, and aspirin. He did not develop any alcohol withdrawal symptoms. His sodium, blood pressure, and mentation improved on day two of admission. Endocrinology was consulted. Sodium improved to 136 mmol/L on day four. His antiphospholipid antibody panel for anticardiolipin, phosphatidylserine, beta II glycoprotein antibodies, HIV antibody, and blood and urine cultures were negative. Drug-induced hyponatremia from HCTZ and metoprolol is possible, but the presentation is extremely severe, and he was on these medications for a few years without a change in dosages. We also ruled out metastasis to adrenal glands. We started him on prednisone 10 mg daily and fludrocortisone 0.05 mg daily on day four of admission, and he was discharged home.

At a 4-month follow-up, an ACTH stimulation test was done after stopping prednisone and hydrocortisone for two days, which showed basal cortisol of < 1 mcg/dL followed by 1.4 mcg/dL and 1.7 mcg/dL confirming no return of adrenal function. We did not check 21-hydroxylase antibody levels (Addison’s disease) as the etiology is obvious with adrenal hemorrhage, and he has no other associated autoimmune diseases. He continued to be on oral steroids, and we resumed his aspirin, atorvastatin, and metoprolol with stable blood pressure and pulse rate.

## Discussion

Bilateral adrenal hemorrhage is a relatively rare disease with many clinical presentations. It is commonly associated with severe sepsis, anticoagulation use, and postoperative stress and trauma. It is associated with a high mortality rate of about 15%. The most common symptoms of adrenal hemorrhage are vague abdominal pain, altered sensorium, hypotension, fever, and significant hyponatremia. The adrenal gland is supplied by three suprarenal arteries and a single adrenal vein, and it also has extensive subcapsular venous plexus. The presence of high vascularity within the gland predisposes vascular congestion and hemorrhage during times of severe stress [9].

Bleeding into the adrenal glands can be unilateral or bilateral. Common laboratory findings are elevated leukocytosis, anemia, and severe hyponatremia. Early identification, aggressive treatment, intensive monitoring, and work-up for underlying risk factors may help decrease potentially life-threatening outcomes. Treatment should be based on the presence of adrenal insufficiency and hemodynamic stability. Our patient had bilateral adrenal hemorrhage, severe hyponatremia, confusion, and leucocytosis but no anemia. Postoperative hypotension associated with hyponatremia suggests adrenal insufficiency and should be worked up for adrenal hemorrhage [1]. Unexplained shock in anticoagulated patients should be worked up for adrenal hemorrhage. 

Hyponatremia in adrenal sufficiency is likely secondary to corticoid and mineralocorticoid deficiency, causing decreased adequate circulating blood volume and hemodynamic changes resulting in elevated anti-diuretic hormone (ADH). Evaded ADH will impair the ability to dilute the urine by limiting the delivery of tubular fluid to the diluting site in the kidney [10]. All patients with adrenal hemorrhage should undergo an ACTH stimulation test to confirm the diagnosis once they are stable as an outpatient [11]. 

Continuous intravenous hydrocortisone infusion is favored over intermittent bolus in treating acute adrenal crises during stressful situations like surgery, sepsis, and septic shock [12]. Conservative management with close monitoring is now preferred over the surgical approach for adrenal hemorrhage [13]. Treatment should focus on hemodynamic stabilization and correcting underlying adrenal insufficiency. Complicated adrenal hemorrhage with retroperitoneal bleeding resulting in shock, requiring multiple transfusions, and failed conservative management should go for interventions like angiography, embolization, and even surgical laparotomy. Surgical intervention has increased morbidity, mortality, and extended hospital stay [14]. 

The prognosis depends on the cause of adrenal hemorrhage, the extent of the hemorrhage, hemodynamic stability, and ongoing infection. Even with appropriate treatment, the mortality rate is nearly 50% in patients with Waterhouse Fredrickson syndrome (bilateral adrenal hemorrhage with severe sepsis). Adrenal hemorrhage may lead to temporary or permanent adrenal insufficiency. Persistent shock and drop in hemoglobin should prompt retroperitoneal hemorrhage and need serial imaging and ICU-level monitoring. Isolated elevation of metanephrines can be seen with recurrent hemorrhage, and follow-up results may show normalization of metanephrines [15]. 

## Conclusions

Adrenal insufficiency should be suspected in all patients with hypotension and hyponatremia, and serum cortisol levels should be checked immediately in all such cases. Treatment should be started based on clinical suspicion and not be delayed for diagnostic confirmation. After initial stabilization, the cause of adrenal insufficiency should be worked up thoroughly.
